# Influence of the Use of Milk Replacers on Carcass Characteristics of Suckling Kids from Eight Spanish Goat Breeds

**DOI:** 10.3390/ani11113300

**Published:** 2021-11-18

**Authors:** Guillermo Ripoll, María Jesús Alcalde, Anastasio Argüello, María Guía Córdoba, Begoña Panea

**Affiliations:** 1 Centro de Investigación y Tecnología Agroalimentaria de Aragón (CITA), Avenida de Montañana 930, 50059 Zaragoza, Spain; bpanea@cita-aragon.es; 2 Instituto Agroalimentario de Aragón (IA2 CITA-Universidad de Zaragoza), 50013 Zaragoza, Spain; 3 Departamento de Ciencias Agroforestales, Universidad de Sevilla, 41013 Sevilla, Spain; aldea@us.es; 4 Departamento de Patología y Producción Animal y Ciencia y Tecnología de los Alimentos, Universidad de Las Palmas de Gran Canaria, 35416 Las Palmas, Spain; tacho@ulpgc.es; 5 Nutrición y Bromatología, Instituto Universitario de Investigación de Recursos Agrarios (INURA), Escuela de Ingeniería Agrarias, Universidad de Extremadura, Avda. Adolfo Suarez s/n, 06007 Badajoz, Spain; mdeguia@unex.es

**Keywords:** rearing system, tissue composition, breed

## Abstract

**Simple Summary:**

Goats are important species due to their contributions to the development of rural areas. Spain has one of the largest goat populations in Europe; however, literature on goat carcasses is very scarce and, therefore, it is crucial to study the specific productive potential of each breed. Previous studies by our team on other Spanish goat breeds have shown some interactions between breed and rearing systems and, consequently, further analysis is necessary. This paper aims to contribute to the general knowledge on the subject.

**Abstract:**

Since goat milk has a higher value than kid meat in Europe, some farmers rear kids with milk replacers, although some studies have stated that kids raised on natural milk yield higher-quality carcasses. Our previous studies showed some interactions between breed and rearing system on carcass and meat quality. This study evaluated the influence of the use of milk replacers on several carcass characteristics of suckling kids from eight Spanish goat breeds (Florida, Cabra del Guadarrama, Majorera, Palmera, Payoya, Retinta, Tinerfeña, and Verata). A total of 246 kids fed milk replacer (MR) or natural milk (NM) were evaluated. Carcass, head, viscera, and kidney fat weights, as well as several carcass measurements (round perimeter, forelimb width, carcass length, forelimb length, and carcass compactness index), were registered. Forelimbs were dissected to study tissue composition. For all studied variables, interactions were found between rearing system and breed. In general, the MR rearing system increased the head and visceral weights, as well as the length measurements and muscle percentages. Conversely, the NM rearing system increased carcass compactness and resulted in higher fat contents, independent of the deposit. The choice of one or another rearing system should be made according to the needs of the target market.

## 1. Introduction

Goats are important species due to their contributions to the development of rural areas [1], provisioning meat and milk, which are among the most valuable services of livestock [2]. Spain ranks second in the European Union in terms of number of goats, producing 20% of the goat milk and 10.9% of the kid meat in the European Union [3]. In addition, the sale of suckling kids makes up 20% of the total income per goat on the dairy farm [4], and 80% of this kid meat originates from the suckling kid category (*cabrito*) [5], whilst the other 20% of meat comes from adult goats that are no longer in dairy production. These suckling kids have a live weight of 6–13 kg and a carcass weight of 3.5–7 kg and are perceived by consumers to be a high-quality meat [6].

Nevertheless, because goat meat has an insignificant position in terms of overall farm production, little attention has been given to it; hence, literature on goat carcass and meat quality is very scarce in comparison with other species. Therefore, it is crucial to study the specific productive potential of each breed.

Eighty-eight percent of European Union goats are raised extensively and slaughtered as kids, with carcass weights between 5 and 11 kg [7]. When kid goats are reared with their dams, the availability of milk for cheese production is decreased. In addition, the milk from goats with feeding kids has less fat and protein than milk from lactating goats without suckling kids [8]. Therefore, some goat farmers remove the kids from their dams at a very young age and rear them with milk replacers. Milk replacers specifically formulated for kids can result in high daily weight gain, but some authors [9,10,11] have pointed out that suckling kids better metabolize nutrients from natural milk, yielding higher-quality carcasses.

Previous studies by our team on other Spanish goat breeds have shown that different carcass and meat quality traits improved when animals were fed natural milk, although some interactions between breed and rearing system were found [10,12,13,14,15]; consequently, further analysis is necessary. The aim of this work was to study the influence of the use of milk replacers on several carcass characteristics of suckling kids from several Spanish goat breeds.

## 2. Materials and Methods

### 2.1. Animals

All procedures were conducted according to the guidelines of Directive 2010/63/EU on the protection of animals used for experimental and other scientific purposes (EU, 2010). Suckling male kids from 8 Spanish goat breeds were used: Florida, Cabra del Guadarrama, Majorera, Palmera, Payoya, Retinta, Tinerfeña, and Verata. The different breeds used have different productive purposes. Palmera and Payoya are clearly oriented to milk production, whereas Guadarrama and Retinta are reared mainly to produce meat, with milk production being a secondary purpose. The others breeds are mainly reared for milk production, with meat production being a secondary purpose. For additional information about breed characteristics and productions, see the Official Catalogue from the Spanish Agricultural Ministry, available at https://www.mapa.gob.es/es/ganaderia/temas/zootecnia/razas-ganaderas/razas/catalogo-razas/ (accessed on 1 January 2021).

All kids were evenly reared at two (FL, MA, PL, PY, and TI) or three farms (GU, RE, and VE) per breed in their respective local areas. Each farm reared approximately half of the kids of one breed into each rearing system. On each farm, kids were randomly selected from those born within a range of 10 days. The age of the dam was not considered, although most of them were between the 4th and 6th lactation. All kids were born from a single parturition and were raised with milk replacers (MR) or natural milk from the dams (NM). Kids of the MR rearing system were fed colostrum for the first 2 days and had free access to milk replacer 24 h a day, which was sucked from a teat connected to a unit for feeding a liquid diet. Commercial milk replacers were reconstituted at 17% (*w*/*v*) and given warm (40 °C). The main ingredients were skimmed milk (≈60%) and whey. The chemical composition of milk replacers was as follows: total fat 25 ± 0.6%, crude protein 24 ± 0.5%, crude cellulose 0.1 ± 0.0%, ash 7 ± 0.6%, Ca 0.8 ± 0.1%, Na 0.5 ± 0.2%, P 0.7 ± 0.0%, Fe 36 ± 4.0 mg/kg, Cu 3 ± 1.7 mg/kg, Zn 52 ± 18.8 mg/kg, Mn 42 ± 14.4 mg/kg, I 0.22 ± 0.06 mg/kg, Se 0.1 ± 0.06 mg/kg, and BHT 65 ± 30 ppm. Kids of the NM rearing system suckled directly from dams with no additional feedstuff. Dams grazed in farm facilities for eight h per day and the rest of the time, they were housed with their kids in a stable. Kids from both rearing systems had free access to water 24 h a day.

The numbers of kids evaluated are shown in Table 1. The 246 kids were slaughtered at an estimated body weight of 8 kg to achieve a target hot carcass weight of 5.0 kg. The night before slaughtering, all animals were transported to the abattoir. Kids had access to water overnight but not to feed and were stalled without access to feed but with access to water. The animals were weighed just prior to slaughter (slaughter weight, SW). Standard commercial procedures according to the European normative of protection of animals at the time of killing (E.U., 2009) were followed. A head-only electrical stunning was applied (1.00 A) to kids, which were then exsanguinated and dressed. Since the traditional method of presenting carcasses in Spain is with the head and kidneys, thoracic viscera were removed and weighed [16], and thereafter, hot carcasses, including the head and kidneys, were weighed. Afterwards, the head was separated and weighed, and then, carcasses were hung by the calcaneus tendon and chilled for 24 h at 4 °C. Dressing percentage (DP) was calculated as HCW × 100/SW.

At 24 h postmortem, kidney fat from the left carcass side was removed and weighed (KF). Then, it was cut with a knife, and the internal colour of the kidney fat was measured using a Minolta CM-2006d Spectrophotometer (Konica Minolta Holdings, Inc., Osaka, Japan) in CIEL*a*b* space (CIE, 1986) with the specular component including 0% UV, observer angles of 10° and 0°, and white calibration. The integrating sphere had a 52 mm diameter, and the measurement area (with a diameter of 8 mm) was covered with a CMA149 dust cover (Konica Minolta Holdings, Inc.). The illuminant used was D65. The spectrophotometer was rotated 90° on the horizontal plane before each reading, and the mean of three readings was used for analysis. The lightness (L*), redness (a*), and yellowness (b*) indices were recorded using SpectraMagic NX software (Minolta Co. Ltd., Osaka, Japan), and the hue angle $\left[h_{ab}=\tan^{-1}\left(\frac{b*}{a*}\right)\cdot \frac{180{}^{\circ}}{\pi }\right]$ and chroma $\left[C_{ab}^{*}=\sqrt[{}]{{\left(a*\right)}^{2}+{\left(b*\right)}^{2}}\right]$ were calculated.

Thereafter, the following carcass measurements were recorded: rump circumference (maximum circumference measurement in a horizontal plane on the hanging carcass), rump width (maximum distance in a horizontal plane at the femur trochanter level), hind limb length (length from the perineum to the distal edge of the tarsus), and internal carcass length (L, length from the cranial edge of the symphysis pelvis to the cranial edge of the first rib) [17]. From these values, carcass compactness (HCW/L) was calculated.

Then, the left forelimb was separated from the carcass in a standardized manner [18,19], vacuum packed and stored at −20 °C until sampling. Once thawed at 4 °C overnight, the forelimb was weighed and dissected into muscle, intermuscular fat, subcutaneous fat, and bone (major blood vessels, ligaments, tendons, and thick connective tissue sheets associated with some muscle), according to Panea, Ripoll, Albertí, Joy, and Teixeira [18]. The tissue composition of the forelimb was expressed as percentages of muscle, subcutaneous fat, intermuscular fat, total fat (intermuscular plus subcutaneous), and bone plus others (tendons, vessels, etc.).

### 2.2. Statistical Analysis

All statistics were calculated using XLSTAT statistical package v.3.05 (Addinsoft, New York, NY, USA). Studied variables were analysed using the ANCOVA procedure with the breed (B) and the rearing system (RS) as fixed effects and the hot carcass weight (HCW) as a covariate, while farm was considered as nested effect. Least square means were estimated, and differences were tested with the Bonferroni test at a 0.05 level of significance. Principal component analysis (PCA) was performed with the tissue composition variables. Only factors accounting for more variation than any individual type trait (eigenvalue P 1) were retained. A Varimax rotation was applied to the retained components to redistribute the variance among factors to obtain factor pattern coefficients. The resulting rotated factors are considerably less correlated than the original ones, making it easier to interpret the components without changing their explanatory power.

## 3. Results and Discussion

### 3.1. Carcass, Head, Viscera, and Kidney Fat Weights

Table 1 shows the slaughter weight (SW), dressing percentage (DP), head weight, visceral weight, and kidney fat weight (KF) as a function of the breed and rearing system.

There were significant interactions between breed and the rearing system for all variables. Hence, to achieve a hot carcass weight (HCW) of 5 kg, Majorera and Palmera kids fed MR and all Tinerfeña kids were slaughtered with the greatest SW. Conversely, Florida and Verata kids fed NM and Guadarrama kids were slaughtered with the lowest SW. The least square in the table has been adjusted for an HCW of 4.965 kg. Because the HCW was the same for all kids, breeds with greater SW had a lower dressing percentage (DP). Within breeds, Guadarrama and Retinta fed MR had greater DP than their counterparts fed NM (*p* < 0.05), while Majorera, Palmera, and Verata fed NM had greater DP than their counterparts fed MR (*p* < 0.05). Independent of the rearing system, the three Canarian breeds (Palmera, Tinerfeña, and Majorera) presented the lowest DP. In the MR rearing system, Guadarrama presented the highest DP values, whereas in the NM rearing system, Verata presented the highest values, although they were no different from Florida. Current DP agreed with those reported by several authors in similar breeds [10,16,20].

The use of MR increased the head weights (*p* < 0.05) of Majorera, Palmera, and Payoya, while the other breeds were not affected by the rearing system (*p* > 0.05). Verata from the two rearing systems had the lightest head, while Majorera fed MR had the heaviest head.

The use of MR increased the viscera weights (*p* < 0.05) of Florida, Payoya, and Retinta, while the other breeds were not affected by the rearing system (*p* > 0.05). Verata reared on both systems had the lightest viscera.

According to the results of this study, the influence of the rearing system is clearly conditioned by the breed but there is no pattern associated with the dairy or meat-production aptitude of the breeds used, that is, the differences are due to the breed and not to its usefulness.

Differences in dressing percentage and head and visceral weights between breeds of suckling kids have been reported previously [15,16], although some inconsistencies can be found in the literature. Since dressing percentage is mainly affected by the weight of the digestive tract [21], some authors have reported that natural milk increases the ruminal and intestinal weights in lambs because these lambs have a lower rumen functionality [22], whereas other authors reported opposite findings, supporting our results [23]. Panea, Ripoll, Horcada, Sañudo, Teixeira, and Alcalde [16] and Perez, et al. [24] found that the use of milk replacer did not affect the DP and head weights of Creole, Malagueña, and Murciano-Granadina suckling kids, but other studies reported that the visceral weight of Murciano-Granadina changed with the rearing system [16].

The KF weight increased when animals were fed NM in Florida, Guadarrama, and Retinta breeds, without changes in the other breeds. This increase is especially noticeable in the Guadarrama breed. Florida from NM system presented the highest KF weights whereas Payoya from MR presented the lowest values, although without differences with Payoya fed NM, Palmera fed MR, and Guadarrama fed MR. The effect of rearing system on fat carcass content agreed with the results from other authors [10,25] as well as the fact that in natural milk feeding regimes, dairy breeds presented higher fat amounts than meat-specialized breeds [10,16].

### 3.2. Kidney Fat Colour

**Figure 1:**
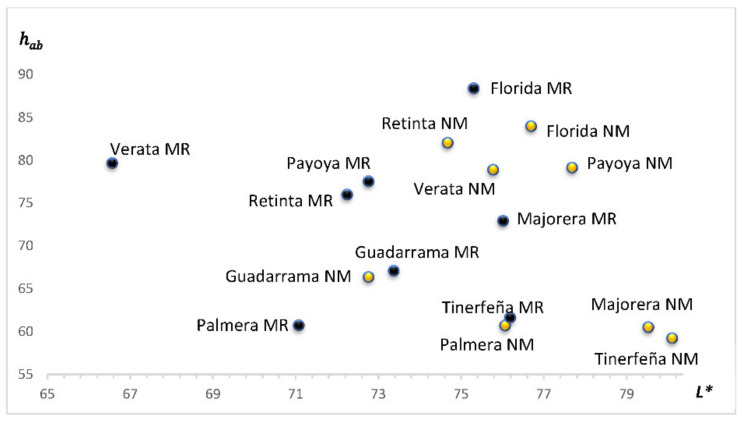
L* versus H_ab_ colour parameters of the kidney fat of kids reared with milk replacer (MR) or natural milk from their dams (NM).

The colour of kidney fat is not a quality cue per se because it is not an eaten fat, but since goats have very little subcutaneous fat, kidney fat is a good option to measure the influence of the rearing system on fat colour. Therefore, there were statistical interactions between the rearing system and the breed for all colour variables (*p* < 0.001). Colour L* and H_ab_ parameters are depicted in Figure 1. In general, L* values were higher for the NM rearing system, although no differences between rearing systems were detected in FL and GU. The $h_{ab}$ was affected only in the Florida and Majorera breeds, with MR values higher than those of the NM breeds.

### 3.3. Carcass Measurements

Means for carcass measurements are presented in Table 2. There were significant interactions between effects for all studied variables (*p* < 0.005). Both the round perimeter (RP) and the hind limb width (LWI) were affected by the rearing system only in the Majorera breed, with NM presenting higher values than MR. Florida presented the lowest values for both variables, independent of the rearing system, whereas Verata presented the highest values. The use of MR resulted in longer carcasses in the three Canarian breeds (Majorera, Palmera, and Tinerfeña), without influence on the other breeds. Majorera, Payoya, and Palmera presented lengthier carcasses than the other breeds, especially in animals from the Payoya breed fed NM. The rearing system affected forelimb length only in Majorera and Tinerfeña breeds, with NM values lower than MR values. The Payoya breed presented a longer forelimb, whereas Palmera and Tinerfeña presented shorter forelimbs. Finally, the rearing system affected the carcass compacity index in all breeds except in Majorera, Payoya, and Verata, with NM presenting higher values than MR.

In general, the MR rearing system increased the length measurements, whereas the NM rearing system increased compactness. Rodríguez, et al. [26] reported greater quality carcasses from kids reared with milk replacers, which agreed with the current results for carcass length and forelimb length.

### 3.4. Tissue Composition

The tissue composition of the forelimb is shown in Table 3. In general, animals fed NM had greater percentages of fat (subcutaneous, intermuscular, and total), while animals fed MR had greater percentages of muscle. Palmera fed MR had the greatest muscle percentage, and Verata fed NM had the lowest (*p* < 0.05). Majorera, Palmera, and Tinerfeña fed NM had the greatest percentages of subcutaneous fat, and Retinta fed MR had the lowest percentage (*p* < 0.05). Florida, Guadarrama, Majorera, Palmera, and Tinerfeña fed MR had the lowest percentages of intermuscular fat, and Tinerfeña and Verata fed NM had the greatest percentages (*p* < 0.05). Florida, Guadarrama, and Payoya had the greatest percentages of bone, and Palmera and Verata had the lowest (*p* < 0.05).

**Figure 2:**
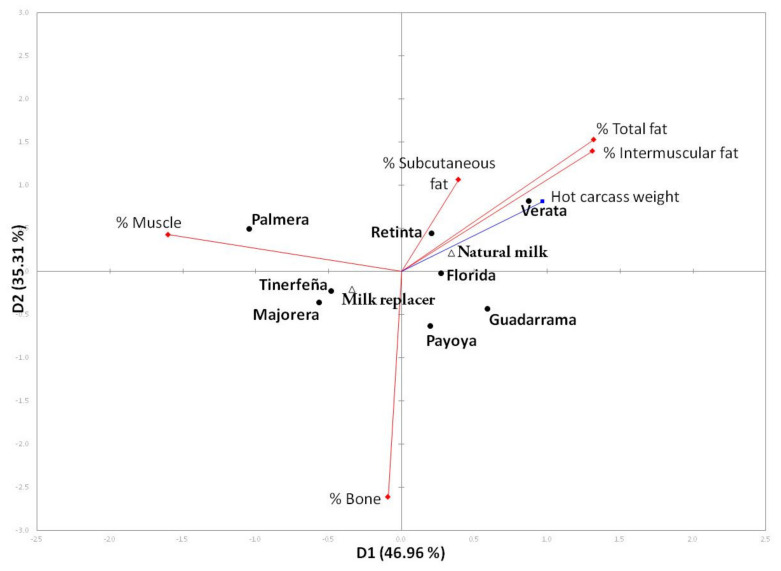
Biplot of the principal component analysis of tissue composition of forelimbs from kids reared with milk replacer (MR) or natural milk from their dams (NM).

The forelimb is often dissected into different tissues because it is easily disjointed, and it is said to be well related to the tissue composition of the carcasses of suckling kids [27,28]. The influence of the rearing system on tissue composition is not conclusive because it is conditioned by breed. De Palo, et al. [29] and Todaro, et al. [30] reported no effect of feed on limb weight or tissue composition, whereas Napolitano, et al. [31] did not find differences in muscle or bone percentages, but forelimb fat was greater when natural milk was used. Similarly, other authors [32,33] reported greater intramuscular amounts when animals were fed natural milk. Finally, Panea, Ripoll, Horcada, Sañudo, Teixeira, and Alcalde [16] reported that natural milk increased the subcutaneous and intramuscular fat percentages of Malagueña, while Murciano-Granadina was not affected by the rearing system. According to our results, the percentages of subcutaneous, intermuscular, and total fat of forelimbs are highly correlated among themselves, but as expected, the higher the bone percentage is, the lower the muscle percentage due to a well-known process called repartition [28,29]. These relationships can be clearly observed in Figure 2.

The usefulness of multivariate analysis to study variable relationships has been demonstrated by several authors [11,34]. The two axes of the biplot explained 82.27% of the variability. The first dimension separates the muscle percentage, on the left, to the fat percentages, on the right. Dimension 2 was explained by the bone percentage. Thus, the three Canary breeds were more muscled than the others, although Verata presented the heaviest carcasses. NM was related to fatness percentages, whereas MR was related to muscle percentage. As expected, muscle percentage was inversely related to fatness degree.

## 4. Conclusions

For all studied variables, interactions were found between rearing system and breed. Hence, farmers should consider the selection of the breed and rearing system together to produce carcasses that the market demands. In general, the MR rearing system increased the head and visceral weights, as well as the length measurements and muscle percentages. Conversely, the NM rearing system increased carcass compactness and resulted in higher fat contents, independent of the deposit.

## Figures and Tables

**Table 1:** Slaughter weight, dressing percentage, and weights of the head, viscera, and kidney fat of kids reared with milk replacer (MR) or natural milk from their dams (NM).

Breed (B)	RS	n	SW (kg)	DP (%)	Head (g)	Viscera (g)	KF (g)
Florida	MR	15	8.0 ^efg^	62.2 ^c^	494.8 ^bcd^	521.6 ^a^	106.7 ^cd^
	NM	15	7.9 ^gh^	62.5 ^bc^	497.3 ^bcd^	447.1 ^b^	196.6 ^a^
Guadarrama	MR	15	7.5 ^h^	66.9 ^a^	504.7 ^bc^	393.0 ^cd^	52.5 ^fg^
	NM	16	7.9 ^gh^	62.4 ^c^	491.3 ^bcd^	376.2 ^de^	148.1 ^b^
Majorera	MR	16	9.5 ^a^	51.2 ^g^	654.3 ^a^	528.9 ^a^	83.1 ^ef^
	NM	16	8.9 ^bc^	54.7 ^ef^	486.6 ^cd^	519.0 ^a^	93.4 ^cde^
Palmera	MR	15	9.2 ^ab^	52.1 ^fg^	505.2 ^bc^	521.2 ^a^	50.7 ^fg^
	NM	16	8.9 ^bc^	55.0 ^e^	451.4 ^ef^	516.9 ^a^	80.3 ^def^
Payoya	MR	16	8.4 ^de^	59.1 ^d^	515.6 ^b^	505.1 ^a^	37.5 ^g^
	NM	14	8.4 ^de^	59.3 ^cd^	474.1 ^de^	419.1 ^bc^	52.6 ^fg^
Retinta	MR	15	7.9 ^fgh^	62.4 ^c^	501.2 ^bc^	348.7 ^e^	63.0 ^efg^
	NM	15	8.3 ^def^	59.2 ^d^	494.7 ^bcd^	271.6 ^f^	156.5 ^b^
Tinerfeña	MR	16	9.2 ^ab^	52.7 ^efg^	501.6 ^bc^	532.5 ^a^	76.8 ^def^
	NM	16	9.3 ^a^	52.4 ^efg^	510.4 ^b^	531.6 ^a^	94.2 ^cde^
Verata	MR	15	8.4 ^dc^	59.7 ^cd^	444.8 ^f^	355.7 ^e^	123.3 ^bc^
	NM	15	7.6 ^gh^	65.4 ^ab^	449.1 ^f^	356.3 ^e^	113.3 ^cd^
	s.e.		0.16	1.09	8.86	12.85	11.65
	B		0.0001	0.0001	0.0001	0.0001	0.0001
	RS		0.1552	0.2817	0.0001	0.0001	0.0001
	B*RS		0.0028	0.0002	0.0001	0.0001	0.0001

B, breed; RS, rearing system; SW, slaughter weight; DP, dressing percentage (HCW*100/SW); KF, kidney fat. s.e., standard error. Least square means were adjusted for an HCW of 4.965 kg. Different superscripts (a,b,c,d,e,f,g,h) indicate significant differences (*p* < 0.05).

**Table 2:** Carcass measurements of kids reared with milk replacer (MR) or natural milk from their dams (NM).

Breed (B)	RS	n	RP (cm)	LWI (cm)	CL (cm)	LL (cm)	CI (g/cm)
Florida	MR	15	29.8 ^h^	8.3 ^f^	40.6 ^cde^	28.8 ^bc^	122.3 ^cd^
	NM	15	30.3 ^gh^	8.2 ^f^	39.0 ^e^	28.4 ^bc^	126.7 ^a^
Guadarrama	MR	15	34.0 ^def^	9.4 ^de^	41.6 ^bcd^	28.7 ^bc^	119.0 ^e^
	NM	16	37.0 ^abcd^	10.2 ^cde^	39.7 ^de^	28.0 ^bc^	124.2 ^ab^
Majorera	MR	16	35.4 ^cde^	9.2 ^ef^	44.7 ^a^	28.6 ^bc^	111.1 ^gh^
	NM	16	39.7 ^a^	10.3 ^cd^	41.7 ^bcd^	20.4 ^de^	117.8 ^g^
Palmera	MR	15	37.4 ^abc^	10.5 ^cd^	43.1 ^ab^	20.4 ^de^	113.9 ^h^
	NM	16	38.7 ^ab^	10.0 ^cde^	40.9 ^cd^	20.5 ^de^	120.2 ^fg^
Payoya	MR	16	34.9 ^cdef^	10.6 ^c^	43.1 ^ab^	30.5 ^a^	114.8 ^ef^
	NM	14	35.9 ^cbde^	10.2 ^cde^	43.1 ^ab^	29.7 ^ab^	115.0 ^ef^
Retinta	MR	15	32.1 ^fgh^	10.9 ^c^	40.6 ^cde^	27.6 ^c^	121.1 ^ef^
	NM	15	33.2 ^efg^	11.1 ^bc^	40.1 ^de^	27.1 ^c^	122.8 ^c^
Tinerfeña	MR	16	37.7 ^abc^	10.1 ^cde^	42.4 ^bc^	20.9 ^d^	116.1 ^gh^
	NM	16	37.8 ^abc^	10.2 ^cde^	39.1 ^e^	19.2 ^e^	125.7 ^ef^
Verata	MR	15	33.9 ^def^	12.3 ^a^	40.2 ^de^	27.6 ^c^	123.1 ^bc^
	NM	15	36.8 ^abcd^	11.9 ^ab^	40.2 ^de^	27.6 ^c^	123.5 ^c^
	s.e.		0.23	0.10	0.15	0.30	1.44
	B		0.0001	0.0001	0.0001	0.0001	0.0001
	RS		0.0001	0.473	0.0001	0.0001	0.0001
	B*RS		0.006	0.001	0.0001	0.0001	0.0001

RS, rearing system; RP, round perimeter; LWI, forelimb width, CL, carcass length, LL, forelimb length, CI, carcass compactness index. s.e., standard error. Least square means were adjusted for an HCW of 4.965 kg. Different superscripts (a,b,c,d,e,f,g,h) indicate significant differences (*p* < 0.05).

**Table 3:** Means and standard errors for forelimb tissue composition from kids reared with milk replacer (MR) or natural milk from their dams (NM).

Breed (B)	RS †	%M	%SF	%IF	%TF	%B
Florida	MR	66.2 ^bcd^	0.8 ^cdef^	7.7 ^f^	8.5 ^h^	25.2 ^bc^
	NM	64.4 ^de^	1.1 ^bcde^	10.3 ^de^	11.4 ^defg^	24.2 ^bcde^
Guadarrama	MR	63.8 ^fg^	0.8 ^cde^	7.2 ^ef^	8.0 ^efgh^	28.2 ^a^
	NM	64.5 ^de^	2.1 ^a^	9.5 ^def^	11.6 ^defg^	23.9 ^cdef^
Majorera	MR	67.7 ^bc^	0.5 ^ef^	6.0 ^ef^	6.4 ^gh^	25.9 ^cde^
	NM	66.9 ^cde^	1.0 ^bc^	6.8 ^def^	7.8 ^cde^	25.3 ^defg^
Palmera	MR	70.3 ^a^	0.8 ^bcd^	5.0 ^ef^	5.8 ^fgh^	23.9 ^i^
	NM	67.3 ^bcd^	1.4 ^b^	7.8 ^cd^	9.1 ^bcd^	23.7 ^hi^
Payoya	MR	65.1 ^ef^	0.6 ^def^	7.6 ^def^	8.2 ^efgh^	26.7 ^b^
	NM	65.6 ^de^	0.6 ^def^	8.7 ^cd^	9.2 ^cdef^	25.2 ^cdef^
Retinta	MR	67.5 ^bc^	0.4 ^f^	8.0 ^cd^	8.3 ^defg^	24.2 ^fghi^
	NM	64.2 ^ef^	0.7 ^def^	12.7 ^ab^	13.4 ^b^	22.4 ^ghi^
Tinerfeña	MR	68.2 ^b^	0.9 ^bc^	5.9 ^ef^	6.8 ^efgh^	25.0 ^efgh^
	NM	65.9 ^de^	1.3 ^b^	6.9 ^def^	8.1 ^defg^	26.0 ^bcd^
Verata	MR	65.6 ^bcd^	0.9 ^cdef^	11.7 ^bc^	12.5 ^bc^	21.8 ^i^
	NM	62.8 ^g^	1.1 ^bc^	12.8 ^a^	13.9 ^a^	23.3 ^ghi^
	s.e.	0.479	0.146	0.513	0.547	0.466
	B	0.0001	0.0001	0.0001	0.0001	0.0001
	RS	0.0001	0.0001	0.0001	0.0001	0.0372
	B*RS	0.0001	0.0055	0.1734	0.3895	0.0001

^†^ RS, Rearing system; e.e., standard error; % M, percentage of muscle; % SF, percentage of subcutaneous fat; % IF, percentage of intermuscular fat; % TF, percentage of total fat; % B, percentage of bone and other tissues. Least square means were adjusted for an HCW of 4.965 kg. Different superscripts(a,b,c,d,e,f,g,h,i) indicate significant differences (*p* < 0.05).

## Data Availability

Data are available under request.
